# Incidence of Myelodysplastic Syndrome in UK Petroleum Distribution and Oil Refinery Workers, 1995–2011

**DOI:** 10.3390/ijerph13050474

**Published:** 2016-05-06

**Authors:** Tom Sorahan, Nuredin Mohammed

**Affiliations:** Institute of Applied Health Research, College of Medical And Dental Sciences, University of Birmingham, Birmingham B13 9TY, UK; n.i.mohammed@bham.ac.uk

**Keywords:** myelodysplastic syndrome, benzene, prospective cohort study

## Abstract

The incidence of myelodysplastic syndrome (MDS) experienced by cohorts of 16,467 petroleum distribution workers and 28,554 oil refinery workers has been investigated. Study subjects were all those male employees first employed at one of 476 UK petroleum distribution centres or eight UK oil refineries in the period 1946–1974; all subjects had a minimum of twelve months employment with some employment after 1st January, 1951. Observed numbers (Obs) of MDS cases were compared with expectations based on national incidence rates for the period 1995–2011. The overall standardised registration ratio (SRR) was 73 (Obs = 17) in petroleum distribution workers for the age-range 15–84 years, and 77 (Obs = 21) for the age-range 15–99 years. The overall SRR was 81 (Obs = 29) in oil refinery workers for the age-range 15–84 years, and 83 (Obs = 36) for the age-range 15–99 years. More detailed analyses were carried out in terms of year of registration, period from hire, decade of hire, and duration of employment. The overall SRR findings did not provide clear evidence for the presence of an occupational cancer hazard, and provide no support for the hypothesis that low-level benzene exposure has an important effect on the risks of MDS.

## 1. Introduction

In 2012, Schnatter *et al.* reported the findings of an international pooled analysis of nested case-control studies arising from cohort studies of petroleum workers from Australia, Canada and the United Kingdom [1]. These authors found a statistically significant, monotonic association between the risks of myelodysplastic syndrome (MDS) and estimates of cumulative benzene exposure. This association was based on 29 incident cases of MDS (median exposure: 3.4 ppm-years) and 129 control subjects (median exposure: 1.4 ppm-years). There was little evidence of a dose-response effect for acute myeloid leukaemia (AML) and the authors concluded that MDS may be a more relevant health risk for lower benzene exposures.

If the MDS findings from the pooled nested case-control studies are real then excesses should be apparent from simple national comparisons of the cohorts that gave rise to the nested case-control studies, because excesses were reported in about two thirds of the combined cohorts (two upper tertiles of cumulative exposure). The UK element of the nested case-control study was based on a cohort of petroleum distribution workers and this cohort has been updated recently along with a cohort of UK oil refinery workers.

The original cohorts comprised 34,569 oil refinery workers [2] and 23,358 petroleum distribution workers [3]. All these male employees had a minimum period of employment of 12 months in the period 1950–1975; some study subjects were first employed before 1910. The cohorts were re-defined in 1995 so that findings would be relevant to more recent work conditions; the recent cohort analyses have been limited to those 28,554 refinery workers and 16,467 distribution workers first employed after 1 January 1946 [4,5]. The re-defined datasets mainly comprise entry cohorts (workers first employed in the period 1950–1974). The extent of any “survivor population effect” present in the sub-cohort of workers first employed in the period 1946–1949 was judged likely to be modest (such workers would only appear in the study if they remained (survived) in the industry until 1 January 1951).

Follow-up data are now available for deaths (1951–2011) and cancer registrations (1971–2011). This report presents findings for MDS to see whether simple national comparisons support the recent hypothesis that low-level benzene exposures in the petroleum industry may have a discernible influence on the risks of MDS. This analysis was conducted in accordance with the Declaration of Helsinki and the protocol was approved by the University of Birmingham Science, Technology, Engineering and Mathematics Ethical Review Committee (project code ERN_12-0638, 10 July 2012).

## 2. Materials and Methods 

The computer file for the revised cohorts contained dates of birth, work history information (dates of commencing employment, dates of leaving employment, job title in 1975 or last job if left employment before 1975), and follow-up information (dates of death, underlying causes of death, contributory causes of death, cancer registration particulars) for 28,554 oil refinery and 16,467 petroleum distribution workers first employed at one of eight UK oil refineries or 476 petroleum distribution centres in the period 1946–1974. Study subjects had to have at least twelve months employment with some employment after 1 January 1951. Six oil refineries and 403 petroleum distribution centres were located in England and Wales; the remaining two oil refineries and 73 distribution centres were located in Scotland.

The National Health Service Central Register (NHSCR) of the Health and Social Care Information Centre (HSCIC) and the General Register Office for Scotland provided vital status information on the closing date of the survey, 31 December 2011. For those who had died (14,841 refinery workers and 9799 distribution workers), information on the underlying and contributory causes of death was supplied in terms of the contemporaneous revisions of the International Classification of Diseases (ICD); the recorded cause of death was untraced for only 19 deaths in refinery workers (0.1%) and 13 deaths in distribution workers (0.1%). A total of 1467 refinery workers (5%) and 314 distribution workers (2%) had emigrated. A total of 336 refinery workers (1%) and 425 distribution workers (3%) were untraced at the end of follow-up.

Myelodysplastic syndrome does not have its own code in ICD-9. Consequently, national comparisons could only be made for 1995 onwards for England and Wales and 1997 onwards for Scotland. Expected numbers of registrations were calculated from male incidence rates (specified by five-year age-groups, five-year calendar periods and country [England and Wales, Scotland]) applied to similarly-defined arrays of person-years-at-risk (pyr) generated by the data. Workers entered the pyr on 1 January 1995 (or 1 January 1997 for Scottish facilities). They left the pyr on the closing date of the study (31 December 2011), the date of death or the date of emigration, whichever was the earlier date. Untraced subjects and workers who died or emigrated before 1995 (1997 for Scotland) were excluded from the pyr because by definition no cases could be ascertained in these groups of workers. These procedures were carried out using STATA [6] software; calculations were also carried out using the EPICURE software [7] and were found to be identical to two places of decimals. In the first analyses no contributions were made to observed or expected numbers past the age of 85 years, because any study subjects incorrectly classified as traced alive at the end of the study would have a disproportionate effect on the expected numbers for deaths after the age of 85 years. However, given that MDS is typically a disease of old age, all analyses were also carried out for the age range 15–99 years (*i.e.*, censoring at age 100 years).

Overall standardised registration ratios (SRRs) were calculated as the ratio of observed cases to expected cases, expressed as a percentage. The significance of the differences between observed and expected numbers were assessed by means of the Poisson distribution. In more detailed sub-group analyses it was judged more important to seek evidence for any trend (linear component) [8] in the pattern of SMRs (e.g., any tendency for SRRs to increase or decrease with time since first employment). All significance tests were two-tailed.

## 3. Results

Observed and expected numbers of incident cases of MDS (ICD-10:D46) for the age-range 15–84 years are shown in Table 1 for petroleum distribution workers by year of registration, period from hire, decade of hire and duration of employment. There was no suggestion of an overall increased incidence of MDS (Obs 17, SRR 73, 95% CI 43 to 118) and there were no significant trends in any of the four sets of SRRs.

Observed and expected numbers of incident cases of MDS for the age-range 15–99 years are shown in Table 2 for petroleum distribution workers by year of registration, period from hire, decade of hire and duration of employment. There was no suggestion of an overall increased incidence of MDS (Obs 21, SRR 77, 95% CI 48 to 118) and there were no significant trends in any of the four sets of SRRs.

Observed and expected numbers of incident cases of MDS for the age-range 15–84 years are shown in Table 3 for oil refinery workers by year of registration, period from hire, decade of hire and duration of employment. There was no suggestion of an overall increased incidence of MDS (Obs 29, SRR 81, 95% CI 54 to 116). There were no significant positive trends in any of the four sets of SRRs; the negative trend for period from hire approached statistical significance (lower SIRs with longer periods from hire) and the negative trend for duration of employment was statistically significant (*p* = 0.03) (lower SIRs with longer duration of employment).

Observed and expected numbers of incident cases of MDS for the age-range 15–99 years are shown in Table 4 for oil refinery workers by year of registration, period from hire, decade of hire and duration of employment. There was no suggestion of an overall increased incidence of MDS (Obs 36, SRR 83, 95% CI 58 to 115) and there were no significant trends in any of the four sets of SRRs. The negative trend for period from hire approached statistical significance (lower SIRs with longer periods from hire) and the negative trend for duration of employment was of borderline statistical significance (*p* = 0.05) (lower SIRs with longer duration of employment).

There was no suggestion of a major healthy worker effect influencing the overall SRR findings. The SRR for all neoplasms (C00-D48) excluding non-melanoma skin cancer (C44) in the period 1971–2011 and for ages 15–84 years was 105 in distribution workers (95% CI 102 to 108) based on 4344 registrations, and 97 in refinery workers (95% CI 94 to 99) based on 6394 registrations.

## 4. Discussion

This study has found no overall excess of MDS incidence in historical cohorts of UK oil refinery and petroleum distribution workers, in comparison with rates in the general population. The study suffers from limitations, most notably the absence of MDS health outcome data prior to 1995, leading to no data on observed or expected numbers in the first twenty years from hire. It is possible that an MDS effect from benzene exposure is only found in this early period of follow-up and that consequently it has been missed in the current study. The study is also limited (currently) to a single job title per worker, so work histories cannot be converted to meaningful exposure histories, there would be too much misclassification of exposure. This latter limitation means that the detailed analyses carried out (e.g., SRRs by duration of employment, and by period from hire) would not necessarily identify a benzene effect on MDS risks. But if a large proportion of the cohort has suffered an important elevated risk of MDS, then an excess should be apparent in the simple overall SRR findings.

Schnatter *et al.* suggested recently that MDS may be a more important health risk than AML for lower benzene exposures, on the basis of the findings from their International pooled analysis [1]. The overall findings presented in this report suggest this is not the case, at least for these cohorts of UK petroleum distribution workers (the UK component of the international study) and oil refinery workers. There is no reason to believe that benzene exposures in the UK petroleum industries were lower than those in Canada or Australia, but more information on this topic would be helpful. In discussing the leukaemia findings from the Australian Health Watch Study [9] (another component of the international pooled analysis), Doll argued that the very high relative risks reported for benzene exposure cannot be regarded as reflecting causality because the interpretation of the nested case-control study has to be consistent with the findings of the cohort study [10]. In other words, if the nested case-control study has correctly identified that a large proportion of a cohort is at a highly elevated risk of some disease, then an excess for this disease should be apparent in simple national comparisons for the overall cohort. The same argument applies to the novel finding of Schnatter *et al.* [1] that cumulative benzene exposure showed a monotonic dose-response relationship with MDS risk. It will be important to know whether there is any overall excess of MDS based on national (or regional) comparisons in those Canadian and Australian cohorts that also featured in the International study [1]. If national comparisons in one or both of these cohorts find no excess, then the novel finding of Schnatter *et al.* [1] cannot, perhaps, be regarded as reflecting causality. Interpretation would also be assisted by incorporating full work histories into the UK cohort (and other cohorts) such that the exposure assessment methodology used by Schnatter *et al.* [1] could be applied to the full cohort(s). It would then be possible to calculate SRRs by cumulative exposure categories.

It would also be informative to have information on MDS risks in those members of the Chinese benzene cohort with lower levels (say < 10 ppm·year) of benzene exposure [11]. Data on MDS mortality are already available from a US cohort of 2266 workers exposed to benzene in a chemical manufacturing plant [12]. The single death from MDS occurred in the highest (≥25 ppm·year) of the three selected exposure groups; the expected number of MDS deaths in the entire cohort was 0.15. It would be dangerous to draw any conclusion from a single death, but clearly an expansion of this US study to include cancer incidence data would be helpful.

## 5. Conclusions

This study has found no overall excess of MDS incidence in historical cohorts of UK oil refinery and petroleum distribution workers in comparison with rates in the general population and does not support the hypothesis that low-level benzene exposure has a discernible influence on the risks of MDS. 

## Figures and Tables

**Table 1:** Incidence of myelodysplastic syndrome (MDS) (ICD-10:D46) in 16,467 UK petroleum distribution workers, ages 15–84 years, 1995–2011.

Variable Levels	Obs	Exp	SRR	(95% CI)	*p*-Value for Trend
by year of registration					
1995–2000	5	6.34	79	(26 to 184)	
2001–2005	6	7.54	80	(29 to 173)	0.73
2006–2011	6	9.25	65	(24 to 141)	
by period from hire (y)					
1–19	-	-	-	-	
20–29	0	0.64	0	(0 to 576)	
30–39	5	5.69	88	(29 to 205)	0.95
≥40	12	16.80	71	(37 to 125)	
by period of commencing employment					
1946–1949	3	3.53	85	(18 to 248)	
1950–1959	5	8.56	58	(19 to 136)	0.78
1960–1969	9	9.11	99	(45 to 188)	
1970–1974	0	1.94	0	(0 to 190)	
by duration of employment (y)					
1–9	6	7.57	79	(29 to 173)	
10–19	4	6.88	58	(16 to 149)	0.96
≥20	7	8.68	81	(32 to 166)	
Total	17	23.13	73	(43 to 118)	

**Table 2:** Incidence of myelodysplastic syndrome (MDS) (ICD-10:D46) in 16,467 UK petroleum distribution workers, ages 15–99 years, 1995–2011.

Variable Levels	Obs	Exp	SRR	(95% CI)	*p*-Value for Trend
by year of registration					
1995–2000	6	6.96	86	(32 to 188)	
2001–2005	6	8.60	70	(26 to 152)	0.86
2006–2011	9	11.69	77	(35 to 146)	
by period from hire (y)					
1–19	-	-	-	-	
20–29	0	0.65	0	(0 to 568)	
30–39	5	5.91	85	(27 to 197)	0.81
≥40	16	20.70	77	(44 to 126)	
by period of commencing employment					
1946–1949	5	4.81	104	(34 to 243)	
1950–1959	6	10.56	57	(21 to 124)	0.61
1960–1969	10	9.87	101	(49 to 186)	
1970–1974	0	2.00	0	(0 to 184)	
by duration of employment (y)					
1–9	7	8.75	80	(32 to 165)	
10–19	6	8.38	72	(26 to 156)	0.99
≥20	8	10.12	79	(34 to 156)	
Total	21	27.25	77	(48 to 118)	

**Table 3:** Incidence of myelodysplastic syndrome (MDS) (ICD-10:D46) in 28,554 UK oil refinery workers, ages 15–84 years, 1995–2011.

Variable Levels	Obs	Exp	SRR	(95% CI)	*p*-Value for Trend
by year of registration					
1995–2000	12	10.36	116	(60 to 202)	
2001–2005	7	11.58	60	(24 to 125)	0.27
2006–2011	10	13.82	72	(35 to 133)	
by period from hire (y)					
1–19	-	-	-	-	
20–29	2	0.71	282	(34 to 1018)	
30–39	5	4.82	104	(34 to 242)	0.08
≥40	22	30.24	73	(46 to 110)	
by period of commencing employment					
1946–1949	3	4.87	62	(13 to 180)	
1950–1959	16	22.67	71	(40 to 115)	0.26
1960–1969	8	5.90	136	(59 to 267)	
1970–1974	2	2.32	86	(10 to 311)	
by duration of employment (y)					
1–9	17	14.97	114	(66 to 182)	
10–19	6	6.11	98	(36 to 214)	0.03
≥20	6	14.68	41	(15 to 89)	
Total	29	35.76	81	(54 to 116)	

**Table 4:** Incidence of myelodysplastic syndrome (MDS) (ICD-10:D46) in 28,554 UK oil refinery workers, ages 15–99 years, 1995–2011.

Variable Levels	Obs	Exp	SRR	(95% CI)	*p*-Value for Trend
by year of registration					
1995–2000	13	11.47	113	(60 to 194)	
2001–2005	9	13.62	66	(30 to 125)	0.34
2006–2011	14	18.29	77	(42 to 128)	
by period from hire (y)					
1–19	-	-	-	-	
20–29	2	0.71	282	(34 to 1018)	
30–39	5	4.96	101	(33 to 235)	0.12
≥40	29	37.72	77	(51 to 110)	
by period of commencing employment					
1946–1949	5	6.77	74	(24 to 172)	
1950–1959	21	28.00	75	(46 to 115)	0.42
1960–69	8	6.28	127	(55 to 251)	
1970–1974	2	2.37	84	(10 to 305)	
by duration of employment (y)					
1–9	18	17.41	103	(61 to 163)	
10–19	10	7.80	128	(61 to 236)	0.05
≥20	8	18.18	44	(19 to 87)	
Total	36	43.39	83	(58 to 115)	
